# Within-Home versus Between-Home Variability of House Dust Endotoxin in a Birth Cohort

**DOI:** 10.1289/ehp.7632

**Published:** 2005-07-05

**Authors:** Joseph H. Abraham, Diane R. Gold, Douglas W. Dockery, Louise Ryan, Ju-Hyeong Park, Donald K. Milton

**Affiliations:** 1 Harvard School of Public Health, Boston, Massachusetts, USA; 2 Channing Laboratory, Department of Medicine, Brigham and Women’s Hospital and Harvard Medical School, Boston, Massachusetts, USA; 3 Johns Hopkins Bloomberg School of Public Health, Baltimore, Maryland, USA; 4 Division of Respiratory Disease Studies, National Institute for Occupational Safety and Health, Morgantown, West Virginia, USA; 5 University of Massachusetts Lowell, Lowell, Massachusetts, USA

**Keywords:** dust endotoxin, endotoxin, intraclass correlation, variance components

## Abstract

Endotoxin exposure has been proposed as an environmental determinant of allergen responses in children. To better understand the implications of using a single measurement of house dust endotoxin to characterize exposure in the first year of life, we evaluated room-specific within-home and between-home variability in dust endotoxin obtained from 470 households in Boston, Massachusetts. Homes were sampled up to two times over 5–11 months. We analyzed 1,287 dust samples from the kitchen, family room, and baby’s bedroom for endotoxin. We fit a mixed-effects model to estimate mean levels and the variation of endotoxin between homes, between rooms, and between sampling times. Endotoxin ranged from 2 to 1,945 units per milligram of dust. Levels were highest during summer and lowest in the winter. Mean endotoxin levels varied significantly from room to room. Cross-sectionally, endotoxin was moderately correlated between family room and bedroom floor (*r* = 0.30), between family room and kitchen (*r* = 0.32), and between kitchen and bedroom (*r* = 0.42). Adjusting for season, the correlation of endotoxin levels within homes over time was 0.65 for both the bedroom and kitchen and 0.54 for the family room. The temporal within-home variance of endotoxin was lowest for bedroom floor samples and highest for kitchen samples. Between-home variance was lowest in the family room and highest for kitchen samples. Adjusting for season, within-home variation was less than between-home variation for all three rooms. These results suggest that room-to-room and home-to-home differences in endotoxin influence the total variability more than factors affecting endotoxin levels within a room over time.

Endotoxin is biologically active lipopolysaccharide, a component of the outer cell membrane of gram-negative bacteria. Endotoxin has potent proinflammatory effects that have been well characterized at high doses among adults but are less well understood in home settings and among infants and children (Liu 2002; Reed and Milton 2001). Data suggesting that early-life infections and exposure to a farming lifestyle decrease the risk of childhood allergic disease have led to the hypothesis that early-life household endotoxin exposure may be protective against asthma and allergy (Braun-Fahrlander et al. 2002; Celedon et al. 2002; Martinez and Holt 1999; von Mutius et al. 2000). To test this hypothesis, epidemiologists have begun to measure endotoxin levels in house dust samples in population-based studies (Braun-Fahrlander et al. 2002; Gehring et al. 2001a, 2001b; Park et al. 2001; Rizzo et al. 1997; von Mutius et al. 2000).

In principle, the goal of endotoxin assessment for use in studies of endotoxin and chronic disease onset is to estimate subjects’ exposure, appropriately integrated over time and space. However, the appropriate sampling strategy is not well defined, and practical limitations often dictate actual sampling protocols. Commonly, endotoxin sampling would include collection of dust at only one point in time from one or at most a few rooms. Repeated endotoxin sampling within the time period of interest is seldom attempted. For optimal classification of chronic exposure, however, the relationship between point exposure measurements and temporal and spatial averages is of particular relevance. Nevertheless, few data are available on the relationship of endotoxin measured at a specific time and place in the home to endotoxin measured in other rooms within the home and at other times.

In the setting of linear regression, the within- to between-subject variance ratio is an estimate of the signal-to-noise ratio and has been used to assess the misclassification bias that occurs when using an imperfectly measured or surrogate exposure. A large within- to between-subject variance ratio indicates that a single exposure sample will provide a less precise estimate of chronic exposure. Park et al. (2000) applied a variance components model to estimate within- and between-home variances in endotoxin measurements in monthly samples over 12 months in a convenience sample of 20 Boston homes. Within-home variations in endotoxin levels were greater than between-home variations, except for endotoxin sampled in bed dust of adult participants. If generally true, the findings of Park et al. (2000) indicate that comparisons of exposure between homes based on a single assessment of endotoxin levels in sites other than the bed are not particularly useful—even for assessing average exposure over 1 year. Sites other than the bed may be relevant for endotoxin exposure, particularly for infants and toddlers in the United States, who often have relatively little dust in their plastic-covered bed mattresses and who spend a great deal of time in other rooms and crawling on the floor. Two recent reports of variability within and between homes in larger epidemiologic studies in Germany suggest that over 1 year, single measurements may be sufficient to distinguish exposure between homes, but that more measurements are needed to assess long-term average exposure (Heinrich et al. 2003; Topp et al. 2003).

In this report, we used a variance components analysis to reexamine the utility of endotoxin measurements in dust collected from different rooms in distinguishing average exposure during the first months to 1 year of life using a sample of 470 homes of children in Boston, Massachusetts. We assessed the correlation of endotoxin sampled in one room with levels in other rooms, and the correlation of a single endotoxin measurement with measurements of endotoxin in the same room 5–11 months later. We estimated room-specific within- to between-home variance ratios and explored the implications of these variance estimates for epidemiologic studies of dust endotoxin and health outcomes.

## Materials and Methods

### Cohort.

The Epidemiology of Home Allergens and Asthma study is a longitudinal birth cohort study of environmental predictors of development of allergy and asthma among children born to a parent or parents with a history of allergy and/or asthma (Gold et al. 1999). The study is investigating the relationship between indoor allergen exposure and the development of allergy and asthma in early childhood. Between September 1994 and June 1996, women who had given birth at the Brigham and Women’s Hospital in Boston were asked if she or the baby’s father had a history of allergy, hay fever, or asthma. Women answering affirmatively were asked to complete a screening questionnaire. Inclusion criteria included history of allergy, hay fever, and/or asthma in at least one parent, maternal age ≥18 years, English or Spanish speaking, residence in the greater Boston area, and no plans to move in the next year. Infants were excluded for premature birth (< 36 weeks), birth with major congenital or teratologic abnormalities, or admission to the neonatal intensive care unit. Of the 1,405 women who completed the screening questionnaire, 499 mothers (505 children) met inclusion and exclusion criteria and agreed to participate.

### Dust collection and endotoxin assessment.

Within the first 3 months of the index child’s birth, an initial exposure assessment was conducted on the 499 homes of participants (Chew et al. 1998). An exposure assessment was conducted approximately 6 months later in a subset of homes. House dust was collected on a 19 × 90 mm cellulose extraction thimble using a modified Eureka Mighty-Mite vacuum cleaner (Eureka Co., Bloomington, IL). Separate dust samples were collected from the kitchen floor, family room, and the floor of the infant’s bedroom. In the kitchen, the floor under cabinets, around the refrigerator, and under the sink were vacuumed for 5 min. In the family room, the seat cushion, arms, and back of the chair most often occupied by the primary caregiver were vacuumed for 2.5 min. Two square meters of the floor surrounding this chair was also vacuumed for 2.5 min. In the bedroom, 2 m^2^ of floor surrounding the baby’s crib was vacuumed for 5 min. Collected dust was immediately placed in airtight bags. Initial sampling of dust to be used for endotoxin analysis was conducted between November 1994 and October 1996. The second dust sampling was conducted in a subset of homes between June 1995 and October 1996. Homes were selected for repeat sampling if the initial sampling was conducted during winter months. In the laboratory, dust samples were sifted using a 425-μm mesh sieve to remove large debris (e.g., breakfast cereal) and provide a more uniformly mixed, fine dust sample for partition into aliquots for several assays. The fine dust was then weighed and aliquoted for future analysis. Dust samples were stored desiccated at −20°C until extraction. Samples were analyzed for allergen and fungi and additionally analyzed for endotoxin only if there was > 200 mg of dust in the sieved sample. Endotoxin levels were not determined for 29 (6%) of the 499 participating homes. Up to six samples (three rooms with up to two samples) were possible per home. In the 470 homes with at least one endotoxin sample, we collected a mean of 2.7 and median of three samples per home.

The endotoxin activity of dust samples was determined with the kinetic *Limulus* assay with the resistant-parallel-line estimation (KLARE) method (Milton et al. 1992, 1997). *Limulus* amebocyte lysate was supplied by BioWhittaker (Walkersville, MD), and control standard endotoxin was obtained from Associates of Cape Cod (East Falmouth, MA). Endotoxin measurements were adjusted for lot-to-lot variation in *Limulus* amebocyte lysate sensitivity to house dust endotoxin [lot 6L016C used for assay of 42% of the samples was used as the standard lot, and nine additional lots each used for 2–11% of samples were adjusted using data from previously described lot-to-lot comparison assays (Milton et al. 1997)]. Control standard potency was determined for each combination of lysate and standard with reference to the reference standard endotoxins EC5 or EC6 [U.S. Pharmacopoeia, Inc., Rockville, MD; 1 ng EC5 and EC6 = 10 endotoxin units (EU)] available at the time the assays were performed, by simultaneous assay of the control with the reference or with a control traceable to assay with the reference. Results are reported as EU per milligram of dust sampled. The median coefficient of variation of the assay of house dust samples, 23%, was previously reported (Milton et al. 1997). None of the samples was below the limit of detection.

### Statistical analysis.

We used SAS version 8.2 for all statistical analyses (SAS Institute Inc., Cary, NC) and assessed the normality of endotoxin distributions using the Shapiro-Wilk normality test. The dust endotoxin data were log-transformed to normalize the distribution of residuals in the mixed-effects models. We compared means in a mixed-effects model to account for correlation of samples within the same home. The correlation of endotoxin measured in dust sampled from different rooms in a home was assessed using Pearson correlation coefficients (with room-specific averages for rooms with replicate endotoxin observations) and using a mixed-effects model.

We fit a mixed-effects model of log endotoxin levels as a function of room and season adjusting for the correlation of repeated measurements within the same home. Inclusion of a random room effect within homes and declaration of a repeated-measures structure allowed us to characterize variation within and between homes and over time (Hamlett et al. 2003; Lyles et al. 1997; Rappaport 1991; Rappaport et al. 1995; Symanski et al. 1996). We obtained parameter estimates using restricted maximum likelihood (Diggle 2002). More precisely, the general mixed-effects model is described by the expression


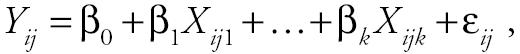


where *Y_ij_* is the *j*th repeated observation of log-transformed endotoxin for home *i*. The terms


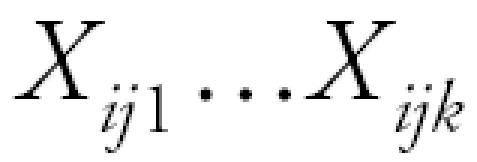


are fixed covariates associated with the *j*th repeated measure on the *i*th home. The residual variance, ɛ *_ij_*, is modeled to include an appropriate correlation structure between endotoxin observations. For each room, the model estimates within-home and between-home variance, σ^2^*_w_* and σ^2^*_b_*, respectively. We then calculated within- to between-home variance ratios and intrahome correlation coefficients for endotoxin sampled from the floor of the subject’s bedroom, the family room, and the kitchen dust samples. The within- to between-home variance ratio characterizes the degree to which a single observation of endotoxin is representative of chronic exposure. The intrahome correlation coefficient characterizes the reproducibility (stability) of repeated endotoxin measurements over time (Rosner 1995). To estimate 95% confidence intervals (CIs), standard errors of within- to between-home variance ratios, and intrahome correlation coefficients were estimated using the delta method, using asymptotic variance and covariance estimates of the room-specific within-home and between-home variances estimated by the mixed effects model.

### Definition of categorical variables.

The season of dust sampling was categorized as winter (November through March), spring (April and May), summer (June through August), or fall (September and October), to match Boston’s climate (Chew et al. 1999). Presence of a pet dog was categorized as no dog versus one or more dogs. The type of house occupied by the family was grouped into *a*) single-family or two-family dwellings or *b*) homes in apartment buildings with three or more units.

## Results

A total of 1,287 endotoxin measurements were taken from 470 of the 499 participating homes. The initial home assessment included 320 bedroom, 401 family room, and 245 kitchen dust samples that were assayed for endotoxin activity. In the follow-up home assessment, endotoxin was measured in 102 bedroom, 147 family room, and 72 kitchen dust samples. In all, 82 (17%) homes had only one endotoxin measurement, 147 (31%) had two measurements, 127 (27%) had three measurements, 61 (13%) had four measurements, 32 (7%) had five measurements, and 21 (4%) had all six measurements. Repeated endotoxin activity measurements were available for 90 bedrooms, 125 family rooms, and 55 kitchens (180, 250, and 110 samples, respectively; Table 1).

### Distribution of endotoxin levels in house dust.

Endotoxin ranged from 2 to 1,945 EU/mg of dust, with a geometric mean (GM) of 82 EU/mg of dust, a median of 81 EU/mg, and a geometric standard deviation of 2.1 (Table 2). Endotoxin was lowest in the bedroom floor samples, intermediate in family room samples, and highest in the kitchen floor samples, and similar in rooms with and without repeat samples. Endotoxin levels were highest during the summer and lowest in winter.

Adjusting for season, home assessment (initial or follow-up), presence of a dog in the home, housing type, and the correlation between observations made in the same home, we found that GM endotoxin varied significantly according to the room in which dust was sampled (*p* < 0.001) (Table 3). Endotoxin levels also varied by season in this model, with highest levels in the summer compared with fall (*p* = 0.002), winter (*p* < 0.001), and spring (*p* = 0.054). Endotoxin did not differ significantly between the initial and repeated samples (*p* = 0.494) in the multivariate model, after adjusting for season. As previously reported, GM endotoxin was higher (*p* < 0.001) in the 75 (16%) homes with dogs relative to homes without dogs, and higher in the 356 (76%) one-and two-family homes compared with homes in multiunit apartment buildings (*p* = 0.004).

### Correlation of endotoxin levels in house dust.

Cross-sectional correlations between room-specific endotoxin levels were low to moderate (Table 4). The mixed-model correlations were similar to the Pearson correlation coefficients. Relative to the cross-sectional comparison of endotoxin from different rooms within homes, repeated room-specific endotoxin levels (5–11 months apart) were more highly correlated for bedroom floor (*r* = 0.65; 95% CI, 0.56–0.75), kitchen floor (*r* = 0.65; 95% CI, 0.53–0.76), and family room (*r* = 0.54; 95% CI, 0.44–0.63). Thus, the temporal correlation of endotoxin levels measured over the 5- to 11-month time was greater than the spatial correlation in those measurements (Table 4). Correlation coefficients estimated without adjustment for season were consistently lower, relative to those estimated while adjusting for fixed effects of season (Table 5).

We divided measurements of endotoxin sampled at two different times into quartiles of the time interval between samples. We did not find any consistent decrease in the correlations moving from shorter to longer time spans between sampling. In fact, for samples taken from the baby’s bedroom floor, the correlation between endotoxin sampled at two points in time increased with increasing time between sampling. However, the sample sizes for each time interval are small and the correlations are correspondingly less stable.

### Endotoxin variance components: variation within and between homes.

We found that season-adjusted within-home variability was lowest for endotoxin in dust sampled from the baby’s bedroom floor, higher in family room samples, and highest for endotoxin in kitchen floor dust (Table 5). Season-adjusted between-home variability was lowest in dust sampled from the family room, higher for bedroom samples, and again highest for kitchen dust endotoxin (Table 5). In models adjusting for season, the within-home variance was less than the between-home variance for all three rooms, suggesting that factors affecting endotoxin levels within a home over time influence the total variability less than factors contributing to home-to-home differences in endotoxin. Within- to between-home variance ratios were lowest for bedroom floor and kitchen floor endotoxin and somewhat higher for family room samples, an observation explained by the comparatively small between-home variation in family room endotoxin (Table 5). These findings suggest that the determinants of endotoxin levels over time and from home-to-home are room specific.

We divided homes into those without dogs and those with at least one dog (Table 6), but found no consistent differences in variance components by presence of a dog. Unexpectedly, we found that between-home variation in endotoxin was uniformly higher in homes without dogs relative to homes with dogs. However, this did not result in correspondingly uniform changes in the correlation of endotoxin levels over time.

A similar subgroup analysis was conducted for housing type (Table 6). We did not find consistent differences in the variance components comparing single- or two-family houses with apartments in buildings with three or more units. Notably, for kitchen endotoxin in multiunit buildings, we observed a within-home variance 3.7 times that of the between-home variance, whereas the within-home variance was smaller than the between-home variance for the other rooms sampled.

## Discussion

We assessed the distributions of, correlations between, and components of variation in endotoxin levels in dust sampled from the homes of subjects participating in an ongoing birth cohort study. In the homes studied, dust endotoxin levels were correlated over 5–11 months (range of intrahome correlations, 0.54–0.65), and slightly less correlated across rooms within homes (range of cross-sectional, room-to-room correlations, 0.30–0.42). Within-home to between-home variance ratios were below one for all samples: 0.53 and 0.54 for bedroom and kitchen dust endotoxin, respectively, and 0.85 for family room endotoxin. Thus, single endotoxin measurements are a reasonable proxy for average exposure during the first few months to 1 year of life and capable of distinguishing among children in metropolitan Boston with regard to endotoxin exposure in early life.

Park et al. (2000) analyzed the variance components of endotoxin in dust collected in a one-year longitudinal study of a convenience sample of 20 homes of students, faculty, and university staff in Boston. This report builds on that work by Park and colleagues by characterizing variability in dust endotoxin using a much larger and more representative sample of homes in the metropolitan Boston area with at most two measurements per room in different seasons. Gereda et al. (2000) made repeated measurements of house dust endotoxin on 11 homes, 6 months apart, finding no significant differences in endotoxin of dust samples from the two assessments. They did not report the replicate data in their limited number of homes. Heinrich et al. (2003) reported repeated measurements of endotoxin in homes over a 1-year period. They found that endotoxin measurements expressed as units per area were more consistent and better able to distinguish between homes than were measurements expressed as units per gram. However, both methods gave higher between- than within-home variances and suggested that single measurements could be used as proxies for average exposure during the year of sampling. In our study, the protocol for collection of kitchen and family room dust samples did not use standard areas because they targeted furnishings or certain architectural features of the rooms, and therefore precluded estimation and analysis of units per area.

Endotoxin levels in a given room were only moderately correlated with those from other rooms in the same home, suggesting that an endotoxin sample from a single room may not indicate endotoxin in other rooms or the house as a whole. In an epidemiologic study, dust sampling in several rooms, plus a determination of the relative time spent by the subject in each room may provide a better estimate of household endotoxin exposure at a point in time. Room-to-room correlations between endotoxin levels within homes were assessed using two approaches: Pearson correlations for simple cross-sectional analysis, using the mean level for each room if repeated measures were available; and a mixed-effects model (Heederik et al. 1991; Rosner 1995). The cross-sectional correlation coefficients are estimates of the degree to which room-specific endotoxin is indicative of endotoxin levels in other rooms within the home. The mixed-effects model estimates correlations using all of the data accounting for the correlation in repeated endotoxin levels and thereby decreasing the uncertainty of these estimates. In this study, the conventional Pearson correlation coefficients were for the most part qualitatively similar to the correlation coefficients estimated from the mixed model.

In our primary analysis, the largest between-home variance component was observed for kitchen dust endotoxin, followed by that for bedroom floor samples. The between-home variance of family room endotoxin was comparatively lower. However, in multiunit buildings, the between-home variation was much lower for kitchen floor samples, compared with the between-home variance for bedroom and family room endotoxin.

Within-home variances were highest for kitchen floor endotoxin. Room-specific differences in the within-home variance component were most dramatic for endotoxin sampled from kitchens in multiunit buildings. The large within-home variation in endotoxin observed for kitchen dust samples may be due to water, food products, and vegetable matter being present to varying degrees in homes and over time.

The reproducibility of repeated endotoxin measurements in dust from the kitchen and bedroom floors, as indicated by intrahome correlation coefficients of 0.65 for both, was greater than reported by Park et al. (2000) The intrahome correlation for endotoxin sampled from the family room (*r* = 0.54) was lower than observed for the other rooms in this analysis but higher than those reported by Park et al. (2000) for kitchen and bedroom floor dust. Park and colleagues did not sample family room dust. Possibly, rooms with more usage and foot traffic have more variability in endotoxin levels over time. The moderate temporal stability of endotoxin levels observed in this assessment suggests that a single exposure assessment provides a reasonable, although not an optimal indication of endotoxin levels over time.

Within-home variability in endotoxin was less than between-home variability for all three rooms, suggesting that factors affecting endotoxin levels within a home over time influence the total variability less than factors contributing to home-to-home differences in endotoxin. We observed smaller within-home variances, larger between-home variances, and correspondingly smaller within- to between-home variance ratios than those observed by Park et al. (2000). In contrast to Park et al. (2000), we sampled dust in a far larger number of homes that were likely more representative of metropolitan Boston area households (e.g., Park and colleagues did not include homes with dogs) and thus also expected to have a larger between-home variance. The present study was limited, however, by having fewer repeated measurements and insufficient repeated bed dust and air samples for analysis. Another limitation was that we could not compute endotoxin loading per unit area because dust was collected from family room furnishings as well as floors and around the perimeters of the kitchen.

The ratio of within- to between-home variance may be used to better interpret reports of associations between endotoxin levels and disease outcomes and inform endotoxin exposure assessment strategies for future studies. In an optimal study of chronic exposure to house dust endotoxin, all variability would be observed between homes and endotoxin levels would not vary over time in the same sampling area. In that case, the within-home to between-home variance ratio would be zero, and provided there are no other sources of bias, a single endotoxin measurement would provide an unbiased estimate of the effects of chronic exposure on an outcome. In practice, a single measurement of endotoxin taken in one room of a home is likely to be an imperfect surrogate for chronic endotoxin exposure in that home. If the within-home variance is nonzero, the observed room-specific endotoxin level will deviate from the true room-specific mean level. If we assume that the observed measure is an imperfect measure of the true mean endotoxin for a room but that the error in measurement is uncorrelated with the true endotoxin level, the association between single samples of endotoxin in homes and health effects is likely to be attenuated relative to the true effect of chronic exposure (Heederik et al. 1991; Zeger et al. 2000).

The relationship between the effect estimate obtained using an observed, misclassified exposure and the true effect estimate has been derived in the univariate setting with one exposure variable and no covariables. The attenuation of the effect estimate is given by


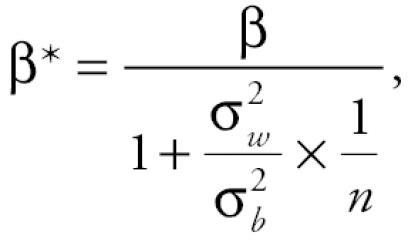


where β* is the observed linear effect estimate, β is the true effect estimate, σ^2^*_*w*_* is the within-home variance, σ^2^*_*b*_* is the between-home variance, and *n* is the number of repeated samples per sampling unit. The magnitude of attenuation increases as the within-home to between-home variance ratio increases. Because the magnitude of misclassification depends not on the value of either within- or between-home variance but on the ratio of the two, there are several theoretical approaches to reduce or avoid the bias of the exposure–outcome relationship. Namely, one could maximize variability of endotoxin across subjects, thereby increasing σ^2^*_*b*_*, or sample endotoxin repeatedly to better estimate true room-specific mean levels within homes.

Applying this theory to our findings, epidemiologic studies using a single house-dust endotoxin observation as an index of chronic exposure may underestimate the effect of endotoxin on an outcome, given that such an effect exists and no other bias or misclassification is present. In the present study, if dust from the three rooms were equally good proxies for actual exposure, using family room endotoxin as the exposure measure, which has the highest within-home to between-home variance ratio, would result in the largest degree of attenuation of effects, relative to using endotoxin from the other rooms.

Variance components provide a statistical basis for sampling but should not be the only determinant of a home sampling strategy. To properly assess exposure, one must consider other determinants of exposure, including where the subjects spend their time. We found that at the time of the first dust sampling, 64% of the children were reported to spend most of their time in the family room, whereas 12 and 6% reported spending most of their time in the kitchen and bedroom, respectively. Eighty-five percent of participants classified the child’s time spent in the family room as more than in other rooms. Thus, use of family room dust samples may provide a better indicator of exposure compared with using only bedroom or only kitchen dust samples.

The true window in which endotoxin exposure may act to modify allergen sensitivity is not known. There is experimental evidence that endotoxin effects are both time and dose dependent (Eisenbarth et al. 2002; Tulic et al. 2000; Wan et al. 2000). It is possible that exposures in a specific perinatal period may be protective of allergic disease development, whereas similar exposures occurring at less relevant periods or at different doses may be innocuous or even promote allergic disease. Thus, the timing of endotoxin exposure sampling with respect to the development of the child may be more important in defining risk than the season in which the sample was collected.

The initial motivation for conducting the repeated-measures dust sampling was to assess the effects of season on indoor allergens focusing on homes initially sampled during winter months. Thus, the second home assessment was conducted in a complementary season relative to the first home visit. The first home assessments were conducted during all seasons, although the samples from homes selected for repeated sampling were collected during the winter and spring months. In contrast, the repeated measurements all were taken from dust collected during the summer and fall months. Because of seasonal variability in endotoxin levels, GM endotoxin was higher for the second home sampling compared with the first assessment. The variability and correlation in endotoxin over time were assessed using both models that adjusted for season of sampling and those that did not. Adjusting for a fixed-effect of season decreased the variability observed within homes and increases variation between homes. As a result, correlations increased and the ratio of within- to between-home variance decreased after controlling for season of sampling.

The endotoxin levels we observed (maximum < 2,000 EU/mg dust) are comparable with those seen in studies of house dust endotoxin in other urban settings (Gereda et al. 2000; Park et al. 2000; Rizzo et al. 1997) but lower than in studies including rural or farm homes (Braun-Fahrlander et al. 2002; von Mutius et al. 2000). We observed lowest endotoxin levels in dust sampled during the winter months and highest levels in dust sampled during the summer. Park et al. (2000) found similar seasonal patterns for outdoor samples of endotoxin but suggested that endotoxin samples from indoor house dust may not follow this pattern consistently. Our finding of seasonal variability is consistent with the findings of Rizzo et al. (1997) in a case–control study of endotoxin and asthma in children 6–16 years of age living in São Paulo, Brazil, who reported endotoxin levels to be generally lower in the winter months and higher in summer months. Study-to-study comparisons of endotoxin are often limited by interlab differences in endotoxin assay protocols. However, the samples described here were assayed by the same laboratory using the same protocols and *Limulus* lysates as the data reported by Park et al. (2000). Because this sample included only urban and suburban homes, our results may generalize only to other metropolitan regions of developed countries in temperate climates. Although we observed variation in endotoxin levels within and between homes in our study, the degree of heterogeneity is likely small relative to industrial or agricultural settings, where sources of endotoxin exist in particular locations and not in others. Similarly, one might expect a larger degree of between-home variability, and perhaps also different patterns of variability in endotoxin if we included both nonfarm and farm households, as has been done in Europe (Braun-Fahrlander et al. 2002).

Our sampling design was not balanced with respect to season, but this poses no problem for estimation of the variance components using the mixed-effects model. The precision of the temporal variance component estimates (within-home variance) was limited by the fact that we sampled endotoxin at most two times from a given room. On the other hand, this sample included a large number of homes compared with previous studies.

All else being equal, bedroom and kitchen floor samples provided slightly more stable estimates of endotoxin over time. Within-home variation in endotoxin levels was smaller than between-home variation for the three sampling locations. The correlation over time and the ratio of within-home to between-home variance observed in this study support the use of a single endotoxin measurement as a marker for chronic endotoxin exposure in association studies.

## Figures and Tables

**Table 1:** Summary of sample sizes for endotoxin in house dust samples.^a^

	No. of endotoxin samples collected			
Assessment	Total	Bedroom floor	Family room	Kitchen floor
Initial	966	320	401	245
Follow-up	321	102	147	72
Combined	1,287	422	548	317
No. of repeated samples	540	180	250	110

a Dust sampling was conducted according to a standardized protocol. Not all homes had sufficient dust collected to assay for endotoxin. In the home with endotoxin observations, the total amount of dust was not associated with endotoxin levels (data not shown).

**Table 2:** Summary of the distribution of house dust endotoxin levels (EU/mg dust) for selected covariates.

					Percentile			
	No.^a^	GM^b^	GSD	Minimum	25th	50th	75th	Maximum
All samples	1,287	82	2.1	2	52	81	127	1,945
Bedroom floor								
Total	422	67	2.0	2	44	67	103	761
Single sample	242	66	2.1	2	43	66	102	761
Repeated samples	180	70	1.8	16	48	70	103	629
Family room								
Total	548	83	2.0	2	53	83	123	1,945
Single sample	298	82	2.1	2	53	83	129	713
Repeated samples	250	83	2.0	14	57	83	119	1,945
Kitchen floor								
Total	317	105	2.2	4	62	110	173	1,201
Single samples	207	101	2.3	4	62	107	166	1,201
Repeated samples	110	112	2.1	12	63	112	191	852
Home assessment								
Initial	966	79	2.1	2	49	77	126	1,201
Follow-up	321	92	2.0	4	59	88	131	1,945
Season								
Summer	458	97	1.9	4	65	97	138	761
Fall	246	83	2.1	9	54	80	120	1,945
Winter	428	69	2.2	2	42	65	110	1,201
Spring	155	79	2.1	9	48	77	135	580
Dogs^c^								
No	1,058	78	2.1	2	49	76	119	1,945
Yes	229	106	2.1	17	68	101	166	956
Housing type^d^								
Single- or two-family	1,001	86	2.0	9	56	86	131	1,249
Multiunit building	286	68	2.4	2	41	66	110	1,945

GSD, geometric standard deviation.

a No. of endotoxin samples collected.

b GMs are unadjusted.

c Presence of a dog in the home was categorized as none versus one or more.

d Housing type was dichotomized as being a one- or two-family home versus part of a multiunit building.

**Table 3:** Fixed-effects results from mixed-effects model.^a^

Fixed-effect variable	Percent change from reference level	95% CI (%)	*p*-Value^b^
Sample			
Bedroom floor	82	76–89	< 0.001
Family room	—		
Kitchen floor	124	112–137	< 0.001
Home assessment			
Initial	—		
Follow-up	96	86–107	0.494
Season			
Summer	—		
Fall	84	75–93	0.002
Winter	69	61–77	< 0.001
Spring	86	73–100	0.054
Dog in home			
No	—		
Yes	131	116–147	< 0.001
House type			
Single- or two-family home	—		
Multiunit apartment	83	73–94	0.004

a Includes fixed effects for room being sampled, home assessment, season, pet dog, and house type. The model provides estimates of the relative change in mean endotoxin for each covariable, independent of the other fixed-effects variables in the model, accounting for the correlation between endotoxin levels measured in the same home. The reference group is endotoxin sampled from the family room during the summer, in single/two-family homes with no dogs. GM endotoxin in the reference group was 98.3 EU/mg.

b Wald test.

**Table 4:** Correlation of endotoxin levels between rooms (off-diagonal) and within rooms over time (diagonal).^a^

	Bedroom floor	Family room	Kitchen floor
Bedroom floor	0.65	0.30	0.42
	—	*n* = 299	*n* = 185
Family room	0.33	0.54	0.32
	*n* = 299	—	*n* = 233
Kitchen floor	0.41	0.27	0.65
	*n* = 185	*n* = 233	—

a Room-specific intrahome correlation coefficients derived from the within- and between-home variance components are presented on the diagonal. Pearson correlation coefficients are below the diagonal and correlation coefficients derived from the variance components are above the diagonal. The mixed-effects model included indicators for fixed effects of season. If replicate samples were available, the average was used to calculate Pearson correlation coefficients.

**Table 5:** Within-home variance (σ^2^_*w*_) and between-home variance (σ^2^_*b*_), the σ^2^_*w*_:σ^2^_*b*_ ratio, and correlations within rooms over time for endotoxin in dust sampled from the bedroom floor, family room, and kitchen floor.^a^

Sample, model	σ^2^_*w*_	σ^2^_*b*_	σ^2^_*w*_:σ ^2^_*b*_(95% CI)	Correlation over time (95% CI)
Bedroom floor				
a	0.038	0.056	0.69 (0.30–1.08)	0.59 (0.48–0.71)
b	0.031	0.058	0.53 (0.26–0.80)	0.65 (0.56–0.75)
Family room				
a	0.050	0.043	1.15 (0.53–1.77)	0.46 (0.36–0.57)
b	0.042	0.049	0.85 (0.45–1.26)	0.54 (0.44–0.63)
Kitchen floor				
a	0.049	0.079	0.62 (0.2–0.99)	0.62 (0.50–0.73)
b	0.045	0.083	0.54 (0.23–0.86)	0.65 (0.53–0.76)

a Variance components for model a were estimated using a mixed-effects model with a random effect for the room being sampled within homes and a fixed room effect. Model b was additionally adjusted for indicators of season.

**Table 6:** Within-home variance (σ^2^_*w*_) and between-home (σ^2^_*b*_) variance, the σ^2^_*w*_:σ^2^_*b*_ ratio, and correlations within rooms over time for endotoxin in dust sampled from the bedroom floor, family room, and kitchen floor, by pet dog(s) and housing type.^a^

Sample homes	Observations (*n*)	σ^2^_*w*_	σ^2^_*b*_	σ^2^_*w*_:σ ^2^_*b*_ (95% CI)	Correlation over time (95% CI)
Bedroom floor					
No dog	353	0.029	0.058	0.50 (0.22–0.78)	0.67 (0.56–0.77)
Dog(s)	69	0.042	0.051	0.82 (−0.52–2.16)	0.55 (0.20–0.89)
One/two-family	325	0.033	0.043	0.76 (0.28–1.24)	0.57 (0.44–0.70)
Multiunit	97	0.024	0.083	0.29 (−0.02–0.61)	0.77 (0.60–0.95)
Family room					
No dog	459	0.045	0.049	0.91 (0.43–1.40)	0.52 (0.42–0.63)
Dog(s)	89	0.021	0.045	0.47 (0.03–0.92)	0.68 (0.51–0.85)
One/two-family	421	0.036	0.038	0.94 (0.40–1.48)	0.52 (0.40–0.63)
Multiunit	127	0.060	0.080	0.74 (−0.02–1.51)	0.57 (0.36–0.79)
Kitchen floor					
No dog	246	0.043	0.082	0.53 (0.13–0.92)	0.65 (0.51–0.80)
Dog(s)	71	0.047	0.068	0.68 (−0.09–1.45)	0.59 (0.37–0.82)
One/two-family	253	0.043	0.079	0.55 (0.18–0.91)	0.65 (0.51–0.78)
Multiunit	64	0.111	0.030	3.72 (−8.10–15.54)	0.21 (−0.16–0.58)

a Variance components were estimated using a mixed-effects model with a random effect for sampling room within homes, a fixed room effect, and a fixed season effect.
